# Systematic Review on the Effects, Roles and Methods of Magnetic Particle Coatings in Magnetorheological Materials

**DOI:** 10.3390/ma13235317

**Published:** 2020-11-24

**Authors:** Siti Khumaira Mohd Jamari, Nur Azmah Nordin, Ubaidillah, Siti Aishah Abdul Aziz, Nurhazimah Nazmi, Saiful Amri Mazlan

**Affiliations:** 1 Malaysia-Japan International Institute of Technology, Universiti Teknologi Malaysia, Jalan Sultan Yahya Petra, Kampung Datuk Keramat, Kuala Lumpur 54100, Malaysia; khumaira_jamari@yahoo.com (S.K.M.J.); aishah118@gmail.com (S.A.A.A.); nurhazimah@utm.my (N.N.); amri.kl@utm.my (S.A.M.); 2 Mechanical Engineering Department, Faculty of Engineering, Universitas Sebelas Maret, Jalan Ir. Sutami 36A, Kentingan, Surakarta 57126, Indonesia

**Keywords:** magnetorheological, smart materials, coating, particle coating

## Abstract

Magnetorheological (MR) material is a type of magneto-sensitive smart materials which consists of magnetizable particles dispersed in a carrier medium. Throughout the years, coating on the surface of the magnetic particles has been developed by researchers to enhance the performance of MR materials, which include the improvement of sedimentation stability, enhancement of the interaction between the particles and matrix mediums, and improving rheological properties as well as providing extra protection against oxidative environments. There are a few coating methods that have been employed to graft the coating layer on the surface of the magnetic particles, such as atomic transfer radical polymerization (ATRP), chemical oxidative polymerization, and dispersion polymerization. This paper investigates the role of particle coating in MR materials with the effects gained from grafting the magnetic particles. This paper also discusses the coating methods employed in some of the works that have been established by researchers in the particle coating of MR materials.

## 1. Introduction

Magnetorheological (MR) material has captured global interests in the smart materials industry and research, due to its alterable functionalities which correspond with the applied magnetic field. The rheological properties of the materials can be varied accordingly with the application of such external stimuli, therefore MR material is considered to be one of the most promising smart materials which can be applied in engine mounts [1], brake systems [2,3,4] and vibration absorbers and dampers [5,6,7], as well as in sensors [8,9,10]. These applications have excellent benefits in industries that involve transportation [11,12], seismic prevention [13], soft robotics [14], and prosthetic legs [15].

**Figure 1:**
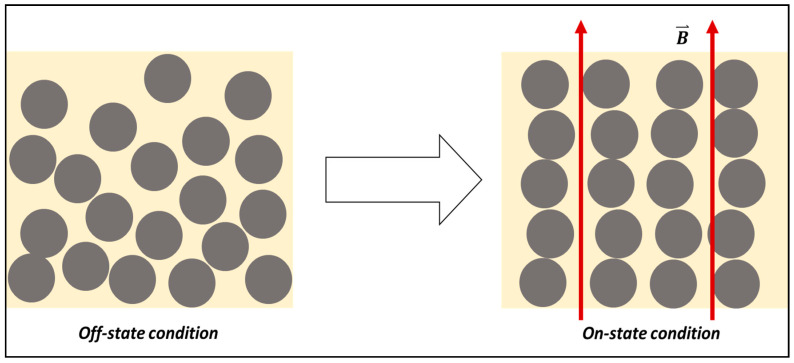
The mechanism of magnetorheological fluid (MRF) prior and upon the application of external magnetic field, $\vec{B}$.

The first MR material was developed by Rabinow [16] in 1948 where a “magnetic fluid clutch” was designed, with a type of magnetizable particles that were mixed in a fluid carrier medium. Under the influence of an external magnetic field, the magnetic particles had been “mutually attracted” and the material “seemingly solidifies”, as mentioned by Rabinow [16]. The fluid was then known as magnetorheological fluid, or MRF, with the most commonly used fluid carriers being silicone oil [4,17,18,19,20,21], synthetic hydrocarbon [22,23,24,25], and mineral oil [26,27]. The mechanism of MRF is as illustrated in Figure 1, where during the absence of an external magnetic field (off-state condition), the material behaves like a Newtonian-fluid, however when the external magnetic field is applied upon the material (on-state condition), the micron-sized magnetizable particles in the fluid align to the direction of the applied field. This causes a restriction of the flow of the carrier fluid, thus changing the behavior of the material to become stiffer and more solid-like. This reversible behavior change can occur almost instantaneously. Due to the chain-like structure of the aligned magnetic particles, yield stress is needed to break the particle chains to allow the flow of the fluid, which means that the higher the applied magnetic field to the material, the stronger the particle chain, and thus the higher the force needed to overcome the yield point where the particle chain begins to deform.

MR grease and MR gel were developed later with a much higher viscosity fluid than MRF to minimize the serious sedimentation problem which occurred in MRF, by reducing the density mismatch between the magnetic particles and the carrier medium. MR grease/gel also has a unique property where it possesses solid gel properties at room temperature, but acts like a fluid at a higher temperature.

Meanwhile, magnetorheological elastomer (MRE) is the solid analogue of MRF, where the fluid is replaced by a non-magnetic elastomer matrix as its carrier medium. By using an elastomeric polymer, such as silicone rubber [28,29,30,31,32], natural rubber [33,34,35] and polyurethane [5,36,37] as its medium, the sedimentation issue in MRF is completely tackled because the magnetic particles are locked within the matrices. It also offers an alternative to the flowy MRF; MRE does not need any vessel to contain it because it is already in a solid form, and therefore there are no leakage or sealing problems in MRE. The particles in MRE are fixed in the network of the elastic polymer, therefore the particles cannot form chain-like structures like MRF when subjected to an external magnetic field, but can only be polarized and mutually attracted to one another, causing a change in the stiffness of the material. The curing process of the material can be done either with or without the presence of an external magnetic field. During the presence of the field, the magnetic particles are aligned in the direction of the magnetic field, causing a columnar particle structure after the sample has cured. This process is called anisotropic curing. Meanwhile, in the isotropic curing method, the magnetic particles are cured and dispersed in no orderly pattern in the MRE due to the absence of a magnetic field. The damping and the storage modulus, as well as loss modulus of these MREs, can be controlled under the influence of various magnitudes of external magnetic fields.

Other less-reviewed MR materials are MR foam and MR plastomer, where the MR foam uses a spongy, absorbent carrier medium such as polyurethane [38,39,40] as its carrier medium. The application of MR foam is also unique; it has been reported that MR foam has a potential application in acoustic absorption [41]. On the other hand, MR plastomer is the latest developed MR material with a carrier medium, such as polypropylene glycol and toluene diisocyanate mixture [42,43], polyvinyl alcohol [44,45], and thermoplastic polyurethane [46], which offers a plasticine-like material that has solid-state between MR gel and MRE. Due to the lack of studies around these forms of MR materials, to the best of our knowledge, there is almost no research involving the incorporation of particle coatings into MR foam and MR plastomer, and therefore in this review, we only focus on the impregnation of coated magnetic particles in the other three MR materials.

## 2. Problems in MR Materials

The advancement of MR materials from one type to another is mainly due to the limitation of one another. For example, because the magnetic particles are much denser than the carrier fluid, sedimentation occurs in MRF during the off-state condition which can lead to another problem—the aggregation of the particles. This aggregation issue may reduce the efficiency of MRF respective to the MR effect that might be reduced over time. Therefore, MR gel/grease was developed to control the particle–medium density mismatch so that it can have a better redispersibility and thus reduce the sedimentation problem. However, because the matrix-based material is highly viscous, MR gel/grease exhibits a high yield stress at the initial state as well as a lower MR effect compared to MRF.

Meanwhile, MRE and MR foam were developed to eliminate the severe sedimentation issue in MRF by embedding the magnetic particles in the elastic and porous matrices, respectively. The leakage and sealant problems in MRF and MR gel/grease has also been overcome by these solid MR materials. However, by locking the magnetic particles in the solid-based matrices, the particles are restricted to move freely and thus inappropriately respond to the applied external magnetic field in the way the particles in MRF do. This affects rheological changes during the off-on state condition in MRE and MR foam, where the MR effect drops several magnitudes in comparison to that of MRF.

Subsequently, the development of MR plastomer may have minimized this MR effect issue, because MR plastomer is reported to have higher MR effect compared to MRE and MR foam, however lower than MRF due to the MR plastomer itself, which exists in the state between both MR materials and possess a high viscosity as well. In addition, the solvent used in MR plastomer may dry up and suffer from a desiccation problem, and therefore it is not suitable for long-term use [44].

Additionally, because the magnetic particles used in MR materials are made up of metal elements such as pure iron and cobalt, oxidation may also take place, especially for long-term use of applications that could further add problems to MR materials. Due to the huge potential that all respective groups of MR materials could offer, progressive research is continuously being undertaken to improve the internal structure of the materials for enhanced properties. Therefore, a particle coating of the magnetic particles in the MR materials has been proposed as one of the ways to tackle the aforementioned problems.

## 3. Particle Coating in MR Materials

**Figure 2:**
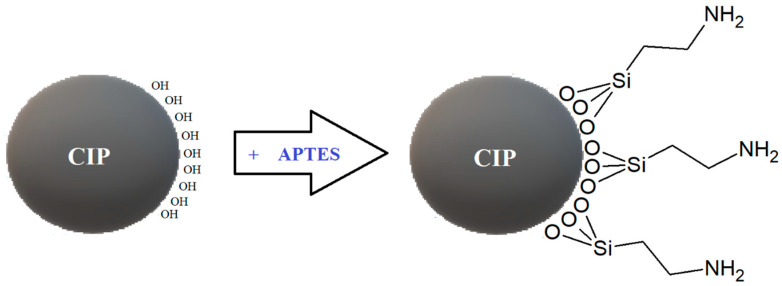
Grafting of (3-Aminopropyl)triethoxysilane (APTES) onto CIP upon activating the surface of the particle with hydroxyl moieties.

**Figure 3:**
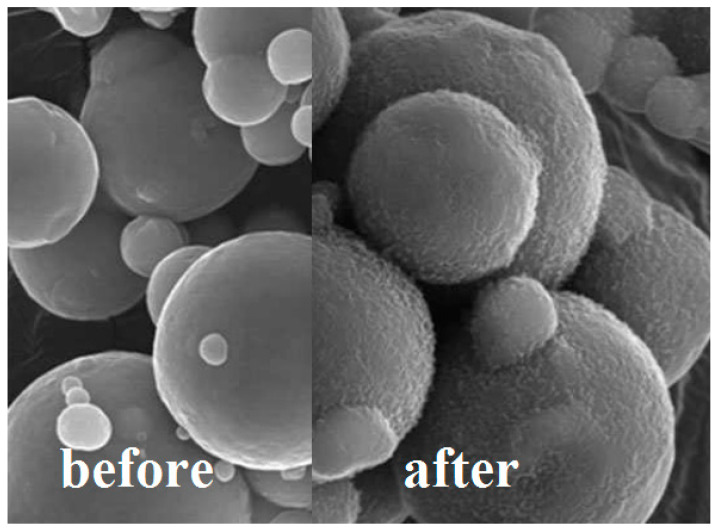
Micrograph of before and after a successful grafting of coating on the magnetic particles (Figure has been modified with reference to [47]).

The advantages of coatings in general are known globally; they can provide extra protection for the substrates that is coated as well as enhance the performance of the materials. The coating application on the surface of magnetic particles has emerged in MR studies, to reduce or minimize the issues that have hindered the full capability of MR materials. This is achieved by grafting a layer of polymer onto the surface of the magnetic particles that later will be incorporated in the MR materials. Figure 2 shows an example of a simple coating onto magnetic particles by grafting (3-Aminopropyl) triethoxysilane (APTES) after the surface of the particle has been pre-treated with hydroxyl moieties. On the other hand, Figure 3 shows an example of a micrograph from scanning electron microscopy (SEM) from a successful grafting of coatings onto the magnetic particles as demonstrated by Sutrisno et al. (2013) [47]. It has been proven that particle coating has great benefits to MR materials in terms of improving the sedimentation stability and re-dispersion of the magnetic particles in MRF, protection against wearing and friction, oxidation protection, and enhancement of rheological properties of the MR materials.

### 3.1. Sedimentation Stability

In a study conducted by Hu et al. (2006) [48], it was found that poly(butyl acrylate)-grafted carbonyl iron particles (CIP) showed a significantly lower settling volume of the magnetic particles in commercial MRF. They also discovered that the sedimentation constant of coated CIP was much lower than that of commercial MRF—the higher percentages of grafted-poly(butyl acrylate) exhibited the lowest sedimentation constant. The team explained that these results are due to the coating layer which acts as a stabilizing layer that can sterically prevent coagulation. Meanwhile, Park et al. (2009) [27] demonstrated that by coating the CIP with poly(methyl methacrylate) (PMMA), the sedimentation problem that occurred in their lubricating oil-based MRF improved due to the lower density of the coated magnetic particles compared to uncoated ones. The re-dispersion of the particles after they were subjected to an external magnetic field was also improved, due to enhanced surface properties such as steric repulsion and electrostatic repulsion of the PMMA coating. A further investigation on the sedimentation stability property of coated magnetic particles in MRF has been conducted by Quan et al. (2014) [49], where a Turbiscan instrument was used to compare the transmission percentage of polystyrene-coated CIP MRF and common silicone oil MRF. At the beginning of the experiment, both MRFs showed zero transmission which represented multiple incident lights scattered through the suspension which could not be transmitted due to many particles that were suspended in both MRFs. However, after 400 min, uncoated CIP MRF exhibited a higher transmission percentage compared to polystyrene-coated CIP MRF, indicating the higher sedimentation stability of the coated sample. The Turbiscan was also used by Tae et al. (2017) [50] to compare the sedimentation ratio of polyaniline (PANI)-coated CIP and pure CIP MRFs by subtracting the transmission ratio from 100% value. In this work, it was found that MRF with PANI-coated CIP exhibited a slower sedimentation speed compared to that of uncoated CIP during the whole 900 min of dispersion period. The team stated that this was due to the thick PANI shell that encapsulated the CIP, causing the density of the particles to reduce significantly. Furthermore, they also claimed that the slightly non-spherical and rough surface of the coated particles also contributed to the improved dispersion stability of the MRF. Moreover, it was also revealed that the time response for coated particles in MRF is faster than that of the uncoated ones, as reported by Nguyen et al. (2014) [4]. In this work, the researchers grafted the CIP with silica to be incorporated in MRF for the application of an MR brake, and it was found that the MRF with silica-coated CIP exhibited a higher settling time of 0.450 s compared to 0.342 s for the same MRF with uncoated CIP.

### 3.2. Tribology Properties

Another significant benefit resulting from particle coating in MRF is related to the unique application of MRF in polishing devices and honing process, as reported by several works [51,52,53,54]. Due to the nature of these works, the magnetic particles used in MRF inevitably experience friction against each other and with the contacting surfaces, which will result in wearing problems of the particles. Furthermore, the use of MRF in dampers and isolators can also cause serious wearing problems to the vessel that contains it [26,55]. Therefore, introducing a coating layer on the surface of the magnetic particles could reduce the fretting problem. It was proven by Bombard and de Vicente (2012) [56], when they compared different grades of commercial CIP in MRF, that it acts as a boundary lubricant between polytetrafluoroethylene (PTFE) and a stainless steel tribopair, which was achieved under a pure sliding contact condition. It was found that OS grade CIP possessed the best friction-reduction behavior compared to the five other grades (OM, OX, HS, HSI and HQ), and even when compared to OM grade that is the same size. This is due to the presence of an amorphous silica coating on the surface of the OS CIP that reduces the friction coefficient of the material and indirectly produces the smallest worn scar diameter in comparison to the other grades. Meanwhile, Zhang et al. (2018) [18] subjected two types of silicone oil-based MRFs to a reciprocating friction and wear test; one was filled with polystyrene-coated CIP and the other one with uncoated CIP. Using a surface profilometer, it was found that the width and the depth of the marks produced by MRF with polystyrene-coated CIP were much narrower and smaller compared to that of uncoated CIP during both off-state and on-state conditions, while the surface of the disc that was coated with CIP MRF was analytically and appeared optically smoother than its counterpart after the wear test. They also discovered that the coefficient of friction of the CIP-coated MRF was lower than that of the uncoated-CIP MRF, especially during the on-state condition. These outcomes undisputedly showed that MRF with polystyrene-coated CIP has better wear and frictional properties than MRF with bare CIP.

In other work conducted by Lee et al. (2015) [57], the CIP was coated with PMMA to be applied in their own MR polishing fluid system. They discovered that by using PMMA-coated CIP in their MRF, the material removal depth of the BK7 glass workpiece was lower while exhibiting lower surface roughness than that of the uncoated one with an optimum rotating wheel speed of 1884 mm/s. This superior surface property of the coated CIP exhibited similar pattern outcomes discovered by the same research team of Lee et al. (2017) [54], but this time, the CIP were coated with a biopolymer, xanthan gum. On the other hand, a study by Hong et al. (2018) [58] also showed the decrease in surface roughness of BK7 glass when the CIP used in their MR polishing fluid was coated with silica, especially when an abrasive was introduced into the MRF. These end results can definitely be utilized in ultra-precision applications in optics and micro parts industries. Another notable outcome achieved by coating the magnetic particles for MR polishing fluid application has been expressed by Salzman et al. (2016) [51], where the CIP was coated with zirconia to be applied as a polishing material on a chemical vapor deposited with zinc sulfide substrate (CVD ZnS). One of the few limitations that hinder MRF from being commercially available for polishing applications is the pebble emergence on the surface of the substrate after the polishing work has been done. The researchers have successfully minimized this problem by using zirconia-coated CIP in their MR polishing fluid in a slightly acidic condition of pH4.

Although there is an increasing number of works which investigate the potential of MRE as a polishing material [59,60], to the best of our knowledge, there is no work that involves coated magnetic particles in MRE for polishing application.

### 3.3. Oxidation

MR materials contain magnetizable particles that inevitably consist of metal elements such as iron, therefore oxidation of the micron-sized particles undoubtedly can occur; this can happen in wet conditions (with the presence of water molecules) or in dry conditions (without the presence of water molecules, which is commonly known as thermo-oxidation).

It has been proven that oxidation phenomena can present negative impacts on the performance of the MR materials, as stated by Lokander et al. (2004) [61]. In their work, the oxidation of the natural rubber of MRE increases as the amount of iron particles increases, due to the oxidation of each individual particle. Furthermore, the high concentration of oxygen elements on the surface of the particles, as well as iron elements of the particles, induces iron ions which are some of the root problems of the oxidation issue of MRE, because the particles are directly in contact with the elastomer matrix. In the meantime, Ulicny et al. (2007) [62] validated the adverse effect of oxidation on MRF after the material was tested for durability performance in an MRF fan clutch. It was found that after 540 h of durability tests, the fan clutch torque capacity reduced gradually as the maximum fan speed experienced dropped by 15%. This is said to be due to the increasing oxidation of the iron particles in conjunction with the fact that iron oxides have much lower magnetization compared to elemental iron. The effects of corrosion in MR materials have also been discussed in detail by Plachy et al. (2018) [17] and Burhannuddin et al. (2020) [63] in separate works around MRF and MRE, respectively. Furthermore, rougher surfaces of oxidized particles may lead to irregular polishing performance as well as reducing the lifetime of the vessels that contain the MR materials, because rougher surfaces can cause unnecessary friction between the particles and the wall of the vessels after a long operational period [57,58,64]. These problems can be minimized by developing coatings that act as a barrier to the oxidation species. It has been proven by Shafrir et al. (2009) [65] in an accelerated corrosion resistance test to determine the oxidation resistance of their zirconia-coated CIP. This test was done by stirring the coated and uncoated CIP in different batches with pH 4.4 acetic acid aqueous solution. After exposure to the accelerated acidic condition for 22 days, the zirconia-coated CIP did not produce any visible goethite (FeOOH) corrosion product. More evidence to the oxidation resistant coating for magnetic particles in MR materials was demonstrated by Cvek et al. (2015) [20], where the team grafted poly(glycidyl methacrylate) (PGMA) onto the CIP to improve the chemical stability of the particles in acidic condition. After exposing the coated and uncoated CIP to 0.05 M HCl in two different containers, the pH values of PGMA-coated CIP remained almost the same throughout the 90 h of acidic exposure, indicating the CIP corrosion protection provided by the PGMA coating. Meanwhile, the performance of coated and uncoated iron particles in MRE before and after an oxidation test has been displayed by Behrooz et al. (2015) [66]. In their study, the poly(tetrafluoropropyl methacrylate)-coated particles and uncoated particles were embedded into silicone rubber MRE. Both samples underwent an accelerated aging test to induce oxidation of the MREs. It was shown that the MRE with coated particles had the least reduction in effective modulus, and the coated particles were able to preserve the stiffness of the MRE under an oxidative environment.

On the other hand, coated magnetic particles in MR materials also exhibited positive outcomes in terms of thermo-oxidation stability. For instance, a facile coating of silicone oil on the CIP by simply immersing the particles in the dimethyl-silicone oil prior being incorporated into MRE, has been proven to enhance the thermal stability as well as the formation of the cross-linked structure of the MRE [67].

### 3.4. Electromagnetic Shielding

One of the most interesting applications of coated magnetic particles incorporated into MR materials is the ability to shield the materials from electromagnetic waves, which can be used in civil and military fields. This can be achieved when core-shell structure particles, which consist of magnetizable particles coated with electrically conducted shell, are able to absorb unfavorable electromagnetic signals and wave pollution that is subjected to the particles [68]. It has been demonstrated that when tetraethylorthosilicate (TEOS) was grafted onto CIP, the MRE that contained these particles exhibited a higher absorbing ability of ultra-high frequencies of 700 MHz to 1.6 GHz, which is the common operation range of communication and information transmission systems. Through this finding, it was claimed that this was the first time that MRE was reported to have the potential to have sufficient electromagnetic shielding [68].

Furthermore, besides the aforementioned advantages of particle coating in MR materials, there are also other benefits that were obtained indirectly through the coating of the magnetic particles into the MR materials’ medium carrier. For instance, in the application of MREs, it was found that the particle’s coating has the capability to promote the interfacial interaction between the magnetic particles and elastomer matrices [69], and this would improve inter-bonding between the particles and the matrix. From the perspective of rheological characterization, it was found that due to the enhancement of the particle–matrix interaction in MRE credited to the grafted coating, the mobility of the particles was enhanced compared to the uncoated particles that were locked within the matrix. This particle mobility reinforcement led to a significant increase in damping property and MR effect of the MRE [70].

From these studies, it can be concluded that particle coating in MR materials can deliver more than one purpose when the coated magnetic particles are dispersed in the medium carrier of the materials.

## 4. Coating Methods

Coating methods employed in particle grafting in MR mainly involve the polymerization of the main monomer on the surface of the particles. This includes Atomic Transfer Radical Polymerization (ATRP), chemical oxidative polymerization, dispersion polymerization and the sol–gel method.

### 4.1. Atom Transfer Radical Polymerization (ATRP)

ATRP is a type of “living”/controlled polymerization that was first discovered by Wang and Matyjaszewski in 1995 as the expansion of the earlier polymerization method, Atom Transfer Radical Addition (ATRA) [71]. However, compared to ATRA, ATRP requires a reactivation of the first formed alkyl halide-unsaturated monomer added, and further reaction of the irregularly formed radical with propagated monomer units. Known for its ease in preparation, ATRP can precisely control the molecular weight of the produced polymer and thus presents the ability to control the thickness of the grafted coat as well as other sequences such as functionalities and architecture [72,73].

One of the advantages that ATRP has in comparison to other controlled polymerization is that it is catalytic, therefore it can be used for a large number of monomers. A very broad range of molar mass chemical substances containing activated (pseudo) halogen atoms can be used as the initiator. ATRP also allows the replacement of terminal halogens with more useful functional groups to be performed rather easily [74]. The polymerization proceeds with irreversible termination and transfer reactions, therefore the polymers obtained can have a predetermined molecular weight and narrow molecular weight distribution [71]. Furthermore, ATRP can also be conducted in mild conditions (i.e., at room temperature) with a high yield [48].

The ATRP process is mainly composed of a monomer, organic halide initiator/retarder, and a catalyst that consists of transition metal species and solvents. Monomers used in ATRP are structural unit compounds that can withstand propagating radicals such as styrenes [47,75,76,77], (meth)acrylates [20,70,78,79,80], acrylates [48,81,82,83] acrylonitrile [84,85] and acrylamides [86,87].

The reaction in ATRP is catalyzed by transition metal complexes that determine the equilibrium constant between the active and dormant species. This means that the catalyst used determines the polymerization rate. However, this equilibrium constant must not be too small, as the polymerization might be inhibited or slowed down, or it could lead to a wide distribution of chain lengths if the constant is too large. Therefore, it is said that the catalyst is the most important component in ATRP because it determines the position of the atom transfer equilibrium and the dynamics of exchange between the dormant and active species [72]. Cuprous salts such as copper (I) bromide (CuBr) and copper (II) bromide (CuBr2) are the most commonly used catalysts in MR ATRP. Although there are a few studies that used copper chloride as the catalyst, the former is used more extensively due to higher reduction potentials compared to the latter, which can be attributed to a stronger Cu–Br bond in comparison to the Cu–Cl bond. These catalysts are complex, with aliphatic amine, imine or pyridine based ligand such as sparteine [47,48,76], bipyridine [79,88,89] or N,N,N’,N”,N”-pentamethyldiethylenetriamine (PMDETA) [70,81,82,87,90,91] which improve its solubility in a polymerization mixture and fine-tune its catalytic efficiency.

**Figure 4:**
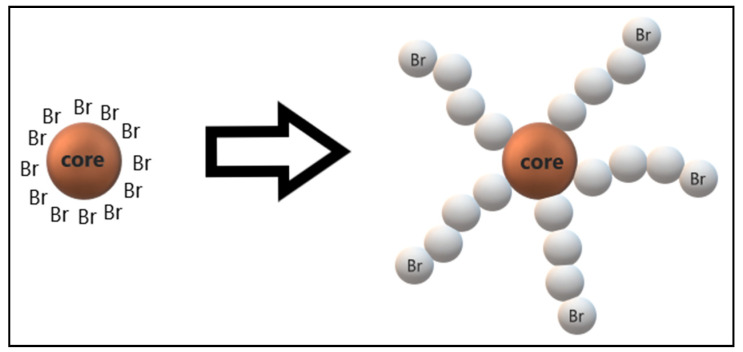
The structure of the bromide initiator influences the star-like architecture of synthesized polymer.

The initiator or dormant propagating chain end, which is usually an alkyl halide (R–X), is included in this type of controlled radical polymerization, where one or more atoms or end groups can be transferred radically. Its role is to react with the monomer to form an intermediate compound. Apart from the monomer conversion, the average molecular weight (Mn) of the synthesized polymers is also dependent on the initial concentration ratio of monomer (M) to the initiator. The halide group (X) must be able to selectively migrate between the growing chain and the transition metal complex rapidly. Therefore, the molecular weight control is the best when bromine or chlorine is used [72]. Commonly used initiators are ethyl α-bromoisobutyrate (EBiB) [20,70,78,81,82,89], α-bromoisobutyryl bromide (BiBB) [70,87,92], methyl 2-bromopropionate [48,75,89] and phenylethyl bromide [75,88]. Additionally, it is noted that there could be more than one initiator used in a single polymerization of ATRP. The number of polymer chains grown for this type of polymerization depends on the type of initiator, because the structure of the initiator influences the architecture of the polymer, as shown in Figure 4.

The solvent is the final component of the ATRP coating method, which is also important in this polymerization, especially when the obtained polymer is insoluble in its monomer such as polyacrylonitrile. ATRP can be conducted in bulk or in solution with the usage of non-polar solvents, such as toluene, xylene and anisole (methoxybenzene) or polar solvents such as dimethylformamide (DMF) [77,81,84,86], acetonitrile [75,81,85] and dimethyl sulfoxide (DMSO) [79,81,84] which are some of the most commonly used solvents in ATRP. However, the type of solvent used must be taken into consideration to minimize the chain transfer to solvent. “Catalyst poisoning” by solvent, which refers to partial or total deactivation of the catalyst due to improper use of solvent must be avoided or at least reduced to ensure efficient ATRP polymerization [93]. Horn and Matyjaszewski (2013) [89] have highlighted the effects of the type of solvent on the activation rate constant with 14 different solvents (anisole, acetonitrile, acetone, butanone, DMSO, DMF, dimethylacetamide, formamide, N-methylpyrrolidone, propylene carbonate, methanol, ethanol, 2-propanol and 2,2,2-trifluoroethanol) which were used to facilitate the polymerization of CuBr/1,1,4,7,10,10-hexamethyltriethylenetetramine (HMTETA) with EBiBB. It was found that the higher the polarity of the solvent, the higher the activation step, while the lower the deactivation step, resulted in a higher the ATRP polymerization constant.

ATRP is one of the most employed coating methods in particle grafting for MR materials. This include two separated works by Cvek et al. (2015) [20,92] where poly(glycidyl methacrylate) (PGMA) was grafted on CIP for the use in MRF. By determining the relative molecular weight using gel permeation chromatography (GPC), relatively narrow polydispersity in both samples were obtained. This indicates that the ATRP process was well controlled and can prevent sedimentation in their MRF samples. Moreover, there was a slight improvement of sedimentation stability in samples which consist of a higher monomer ratio, thus the authors claimed that the length of the polymer chains does not play significant role as expected [92]. This CIP–PGMA sample also exhibited excellent anti-acid corrosion where the coating almost covered the CIP core. However, a small production of hydrogen gas was observed, which indicated that the (3-aminopropyl)triethoxysilane (APTES) base as not fully compact, thus Cl from hydrochloric acid was still able to react with the core [20].

Meanwhile, the grafting of poly(butyl acrylate) was done by Hu et al. (2006) [48] for MRF samples under two separate conditions: a) different butyl acrylate ratio, and b) different reaction time. It was found that the settling was reduced by increasing the coated polymer fraction. The dispersibility of the particles and the sedimentation rate were also greatly improved with the increasing polymer coating fraction. In work done by Sutrisno et al. (2013) [47], poly(2-fluorostyrene) was grafted onto the iron particles for the applications of MRF, while Fuchs et al. (2010) [76] grafted poly(fluorostyrene) onto the iron particles for the applications of MRE. From these works, due to the presence of fluorine, the degradation rate of the coating was low, which implies that it has excellent anti thermo-oxidation property. This has been proven by characterizing the coated samples using differential scanning calorimetry (DSC) and thermogravimetric analysis (TGA). In two separate studies by Cvek et al. (2017) [70] and (2018) [80], the CIP was grafted with poly(trimethylsilyloxyethyl methacrylate) chains to be fabricated in a poly(dimethyl siloxane) (PDMS) MRE matrices. The density of the coated CIP decreased by more than 5% with substantial enhancement of its thermo-oxidation stability. The polymer coating also exhibited extremely stable acidic oxidation, which proved that the grafted layer was uniform without any defects and thus provided an excellent protection against acidic oxidation. Table 1 shows the summary of the ATRP coating method that has been utilized for particle coating in MR materials to date.

With a wide variety of monomers and initiators that can be used in ATRP, there are a lot of options that can be employed to coat the magnetic particles used in MR materials and thus enhance the performance of the materials.

### 4.2. Chemical Oxidative Polymerization

Chemical oxidative polymerization (sometimes called chemical oxidation polymerization) is a detachment process of two hydrogen atoms from monomer molecules to form a covalent bond of a polymer [94]. It is regarded as one of the cleanest and lowest loading methods in poly-condensation or condensation polymerization. Commonly used monomers are aromatic amines, aromatic hydrocarbons, thiophenols, phenols and heterocycles, which have high oxidation tendency due to the presence of electron donor substituents. The oxidation of these monomers is performed under the influence of an oxidizing agent (chemically) or by applied potential (electrochemically). Thus, the polymer growth can be initiated due to the generated cation or cation radical sites of the monomer [95]. Therefore, the main components of this polymerization are the electron-donor monomer, the dopant and the oxidant catalyst (initiator).

A conducting polymer is the most common polymer synthesized from chemical oxidative polymerization. It is used as a corrosion inhibitor [96,97], as electromagnetic wave shielding and an absorbance material [98,99], as well as in other electronic devices and semiconductors [94]. The most researched conducting polymer synthesized via chemical oxidative polymerization is polyaniline. It has been widely adopted in conducting polymer-based composites due to its controllable dielectric loss ability, ease of synthesis, and chemical and environmental stability [97,98,99,100]. Due to its unique interaction with strong acids as its charge stabilizing agent, polyaniline has three main states: pernigraniline, emeraldine and leucoemeraldine, with each of these states existing in a protonated or deprotonated condition. This means that this polymer can exist in at least six different degrees of oxidation and protonation states [94]. Like other conducting polymers, the properties of polyaniline depend strongly on the doping level, protonation level, ion size of dopant and the water content. The emeraldine base form of polyaniline is an electrical insulator consisting of two amines followed by two imines. It can be converted into a conducting form by two different doping processes, which are protonic acid doping and oxidative doping. In the former, the emeraldine base corresponds to the protonation of imines in which there is no electron exchange, while in the latter, emeraldine salt is obtained from leucoemeraldine through electron exchanges. These reversible mechanisms are caused by the presence of –NH groups in the polymer backbone, whose protonation and deprotonation will bring about a change in the electrical conductivity and the color of the polymer [94].

Lin et al. (2017) [100] experimented the polymerization of polyaniline with various polymerization temperature of −7 °C to 60 °C and found that the formation mechanism as well as the structural and electrical properties of polyaniline emeraldine salt synthesized in strong acidic environment are sensitively affected by the temperature. At high polymerization temperature, the development of nanofibers of aniline is limited, thus the granular emeraldine salt samples possess a weaker hardness, less crystallinity, lower molecular weight and a smaller dispersity compared to those synthesized at lower temperatures. Conventional chemical oxidative polymerization of anilines is carried out in an acidic medium (the dopant) and initiated by an oxidant under an ice bath condition. The most commonly used oxidant is ammonium persulfate (also known as ammonium peroxydisulfate, APS), while some works have mentioned the employment of sodium persulfate [101] and potassium persulfate [97].

Some works have reported the associate polyaniline coating in MR application via chemical oxidative polymerization. For instance, Fan et al. (2013) [99] synthesized CIP/polyaniline composites with APS as the oxidant and p-toluenesulfonic acid (p-TSA) as the dopant with different doped acid mole ratio of p-TSA to aniline (0.005:1; 0.05:1; and 0.2:1 ratios). Obvious core-shell structure of polyaniline on CIP was observed using SEM where the coating layer becomes smoother as the doped acid mole ratio of p-TSA to aniline is higher. It was also reported that by observing the permeability and permittivity behavior, it was noted that the impedance matching between the material and the free space had been increased, which showed the capability of the material to absorb microwave radiation. Although in this work no MR application was mentioned, there is still a possibility that this could be implemented in MR materials because the CIP/polyaniline composite is reported to possess excellent microwave absorbing properties.

Meanwhile, Yu et al. (2017) [69] attempted to improve the interfacial interactions between CIP and polyurethane/epoxy elastomer matrix using polyaniline as an interfacial coating, by making use of the presence of amines and imines in the polymer backbone. The attempt was successful, because the polyaniline-coated CIP and the matrix interfacial interactions were improved due to the covalent bond between these components. The contact angles of the polyaniline-coated CIP displayed an angle 24.3° higher, which suggests an excellent hydrophobic property, while there were obvious increments of storage modulus as well as reduced loss modulus compared to uncoated CIP in the elastomer matrix. In the meantime, a study by Tae et al. (2017) [50] showed the importance of surface treatment of the magnetic particles upon coating with a conducting polymer via chemical oxidative polymerization. In this work, the authors demonstrated the attachment of hydroxyl groups on the surface of CIP using p-TSA monohydrate prior to encapsulating the particles with polyaniline to improve the chemical affinity between polyaniline and the CIP. As a result, the polyaniline-coated CIP had better thermal stabilities, while the sedimentation ratio was greatly improved.

In 2011, a study conducted by He et al. (2011) [98] showed that PANI-coated CIP and PANI-coated iron (II, III) oxide (Fe_3_O_4_) particles that were synthesized through chemical oxidation polymerization exhibited good microwave absorbing properties. By plotting Cole–Cole semicircle plots, it was claimed that PANI attributed to the dielectric relaxation process of both coated particles. Furthermore, when both PANI-coated particles were mixed to form a composite of PANI/CIP/Fe_3_O_4_, an appropriate electromagnetic impedance match between these particles was insisted to be the factor that contributes to the enhanced electromagnetic wave absorption of the material. Although the authors did not claim this composite to be used in MR materials, it still has the potential to be applied in this smart material, especially for MRE for electromagnetic wave shielding applications.

To the best of our knowledge, only polyaniline has been associated as the conducting polymer coating for MR materials via chemical oxidative polymerization. However, other conductive polymers that have been reported to be synthesized using this polymerization steps for other applications include polyphenylenediamines, polytoluidine, polypyyrole, polyaminopyridine, polyaminonaphthalene, polyaminoquinoline, polymethylquinoline and polyphenylenediamine, which may open possibilities for ventures into a new type of conductive polymers as the coating layer on the surface of the magnetic particles in MR materials. Some studies of coatings developed via chemical oxidation polymerization in a core-shell structure have been listed below in Table 2 for the potential employment for particle coating application of MR materials. Table 2 includes the type of developed polymers, the type of substrates that were coated onto them, and the findings from the mentioned studies. However, extensive studies must be done to ensure the compatibility of the polymers to be polymerized onto magnetic particles before being incorporated into MR materials, because most of the substrates used are non-magnetic, except [102].

### 4.3. Dispersion Polymerization

Dispersion polymerization as defined by IUPAC (2011) is a precipitation-type polymerization in which monomer(s), initiator(s) and colloidal stabilizer(s) are dissolved in a solvent forming an initially homogenous system that produces polymers and results in the formation of polymer particles [107]. This process usually yields polymer particles in colloidal dimensions. It attracts researchers worldwide due to its ability to form monodisperse particles in a single batch process [108]. One of the unique characteristics of dispersion polymerization is that the solvent used as the reaction medium must be one that has compatibility with the monomers used but incompatible with the polymers that are formed. Therefore, the composition of polymers that are polymerized using this method are usually made from common monomers such as styrenes, methacrylates and vinyls, with a good selection of solvents and initiators.

In the MR field, some works that implemented dispersion polymerization method as the coating method includes the work by Park et al. (2009) [27], where the team encapsulated CIP with PMMA to be used in the MRF. In this work, after the CIP were pre-treated with methacrylic acid (MAA), the particles were dispersed in methanol solution that contained poly(vinyl pyrrolidone) (PVP) that acts as a stabilizer. Then the methyl methacrylate (MMA) monomer was dissolved in the reactor system together with 2,2-azobisisobutyronitrile (AIBN) that acts as radical initiator, as well as ethylene glycol dimethacrylate (EDGMA) that acts as the cross-linking agent to produce cross-linked PMMA during this polymerization steps. A reasonably smooth coating with some surface irregularities was produced, while the sedimentation stability of the MRF was enhanced. A similar work of grafting a PMMA coating onto CIP via dispersion polymerization has also been accomplished by Lee et al. (2015) [57], but with further characterization on the performance of the coated CIP in MRF for the application of optical polishing. It was reported that the sedimentation ratio and anti-corrosion property of the coated material had increased, although the yield shear stress of the coated material in a magnetic field was lower than that of uncoated material.

A core-shell structured polystyrene coated on CIP has been demonstrated by Quan et al. (2014) [49] using dispersion polymerization too. In this work, the researchers also used MAA, PVP and AIBN as the surface pre-treatment agent, stabilizer and initiator, respectively. From the SEM results, it was observed that the coating formed on the CIP surface was not uniform, with granule-like substances that were seen scattered unevenly on the surface of the particles. Despite this, it was reported that the dispersion stability of the MRF that the particle composite had been incorporated too and had been increased, and the shear stress of the material had also been increased. Similarly, Zhang et al. (2018) [18] performed a similar synthesis of CIP/polystyrene in their work but with extended characterization in terms of tribology and the rheological properties of the material. It was noted that the yield shear stress, shear viscosity, and the storage modulus the in MRF that contained the polystyrene-coated CIP was lower than that of that contain uncoated CIP, while the tribology tests showed that the former MRF possessed superior wear and frictional properties than the latter.

Meanwhile, a novel poly(glycidyl methacrylate) coating was synthesized by Seung et al. (2019) [29] on the surface of CIP, which later were embedded in both isotropic and anisotropic silicone rubber-based MRE. In this work, MAA, PVP, AIBN and EDGMA were also used as the pre-treatment agent, stabilizer, initiator and cross-linker, respectively, while glycidyl methacrylate (GMA) was used as the monomer. A rougher but bumpy-like coating was observed in this work. According to the team, the dynamic properties of the MRE that consisted of PGMA-coated CIP had superior dynamic properties with a smaller Payne effect than the MRE that contained uncoated CIP.

From these works, it was noted that in most MR materials that adopted the dispersion polymerization for surface coating method, only the monomer was changed for the polymerization to occur. Thus, there is still room for various types of pre-treatment agent, stabilizer, and initiator, as well as the cross-linking agent to be used in this type of method that can be explored.

## 5. Conclusions

In conclusion, according to the results obtained by studies on particle coating that was then incorporated in MR materials, the coating layer was not only able to protect the magnetic particles from damaging factors such as oxidation, but can also contribute to the enhancement of magnetorheological properties of the material such as the improvement of the sedimentation stability of MRF, increment in wear and friction resistance, as well as reinforcement of the rheological properties of MR materials. It is also emphasized that the coated particles can contribute to more than one advantage that can be offered.

It was clear that almost all MR materials that were impregnated with coated magnetic particles have lower magnetic saturation compared to that of uncoated particles. This is unavoidable because most coatings used are made up from non-magnetic materials such as polymers. However, there were some coated particles that exhibited only a slight drop in magnetic properties, where the materials that were grafted onto the particles were made up from magnetizable materials such as multi-walled carbon nanotubes (MWCNT) [109]. However, when this MWCNT-coated CIP was re-coated with COOH–MWCNT, the magnetic saturation of the particles decreased by about 25%. Therefore, it was ascertained that the thicker the coating layer grafted onto the magnetic particles, the lower the magnetization of the particles, regardless of the type of the coating material.

On the other hand, ascertaining the variety of coating methods that were employed in this MR field may also contribute to the different coating functionalities and properties that can influence the characteristic of the MR materials, with various types of polymers that can be used as the coating layer. Table 3 shows the aforementioned coating methods along with their respective responding variables that are crucial in MR studies in terms of storage modulus, loss factor/loss modulus, MR effect, oxidation stability, sedimentation stability and magnetic saturation as well as other notable outcomes. Some characterizations are unable to be presented into numerical data due to different methods employed by respective authors. Therefore, we only indicated the improvement of the particle coated MR materials characteristics using symbols of ↑ (increase) and ↓ (decrease) when comparing the materials with the uncoated ones. From Table 3, it can be deduced that ATRP is the most commonly used coating method employed in particle coating for MR materials. This might be due to controllable polymerization of ATRP with a wider variety of functionalities that the method could offer, as well as the fact that most MR materials employed in this method have a much lower decrease in magnetic saturation compared to other methods listed in the table below.

Although there are some unavoidable disadvantages when utilizing particle coating in MR materials, such as the lower magnetic saturation, there are still a lot of opportunities for enhancement that can be developed in the near future.

## Figures and Tables

**Table 1:** Summary of atomic transfer radical polymerization (ATRP) coating method employed by some works.

Monomers	Initiators	Catalysts	Solvents	Remarks	Ref.
Glycidyl methacrylate	BiBB EBiB	CuBr PMDETA (L)	THF Anisole Acetone Ethanol	APTES was used as a coupling agent during surface treatment. CIP was pre-treated with HCl.	[20,92]
Butyl acrylate	CTCS	CuBr CuBr2 Sparteine (L)	THF Toluene		[48]
Fluorinated styrene	CTCS	CuBr CuBr2 Sparteine (L)	Toluene Octyl pyrrolidone		[47,76]
2-hydroxyethyl methacrylate Chlorotrimethyl-silane	BiBB EBiB	CuBr PMDETA (L)	Dichloro-methane Anisole	APTES used as a linker of BiBB Et3N used to trap hydrogen chloride	[70,80]

**Table 2:** Some studies that employed chemical oxidation polymerization outside of the magnetorheological (MR) field for coating applications.

Polymerized Polymers	Substrates	Findings	Ref.
Polypyrrole	Sulfur nano-sphere	For rechargeable lithium/sulfur batteries application - Minimize loss of active materials during cycling	[103]
Polypyrrole	Glass beads	For water treatment application - increase adsorption of humic acid - high positive zeta potentials for wide range of pH values	[104]
Poly(1,5-diaminoanthra-quinone)	Carbon cloth	For supercapacitor application - extraordinary cycling stability - ultrahigh capacitance retention (159%) after 20k cycles	[105]
Polythiophene	Nano-silicon	For lithium batteries application - good cycling stability	[106]
Polyrhodanine	γ-Fe_2_O_3_ nano-particles	For antibacterial application - reduction in *Escherichia coli* and *Staphylococcus aureus* colonies (90–99.9%) after contact for 60 min	[102]

**Table 3:** Summary of coating functionalities, classified into coating methods as discussed in the text.

Coating Methods	Ref.	Storage Modulus	Loss Modulus/Factor	MR Effect	Chemical Stability	Thermal Stability	Sedimentation Stability	Magnetic Saturation	Other Characteristics
ATRP	[92]	↑	↑	↑		↑	↑	7.02–9.47 emu/g ↓	
	[76]			↑	↑	↑			
	[47]			↑		↑			
	[70]	↓	↑	↑	↑	↑		6.2% ↓ (~10 emu/g)	↑ hydrophobicity ↑ magnetostriction
	[20]			↓	↑			9–14 emu/g ↓	↑ cytotoxicity ↓ yield stress
	[80]	↓	↑	↑	↑	↑		~20 emu/g ↓	↑ roughness
COP	[50]	↑	↓			TGA: ~90wt% at 800 °C ↑	↑	59 emu/g ↓	
	[110]							42 emu/g ↓	No comparison for other parameters
	[69]	↑	↓ (strain sweep) ↑ (frequency sweep)					31.5 emu/g ↓	↑ hydrophobicity
Dispersion polymerization	[29]	↑	↓	↓				45 emu/g ↓	
	[18]	↓					↑	36 emu/g ↓	↓ yield stress ↓ shear viscosity ↑ friction resistance
	[57]				↑		↑		↓ yield stress ↓ shear stress ↑ material removal depth
	[49]							10 emu/g ↓	↑ dispersion stability ≈ shear stress
	[27]						↑	24 emu/g ↓	↓ shear stress ↓ shear viscosity
